# Antibiofilm Effect of Octenidine Hydrochloride on *Staphylococcus aureus*, MRSA and VRSA

**DOI:** 10.3390/pathogens3020404

**Published:** 2014-05-06

**Authors:** Mary Anne Roshni Amalaradjou, Kumar Venkitanarayanan

**Affiliations:** Department of Animal Science, University of Connecticut, 3636 Horse Barn Hill Road Ext., Unit 4040, Storrs, CT 06269, USA; E-Mail: kumar.venkitanarayanan@uconn.edu

**Keywords:** biofilm, inactivation, *S*. *aureus*, MRSA, VRSA, octenidine hydrochloride

## Abstract

Millions of indwelling devices are implanted in patients every year, and staphylococci (*S*. *aureus*, MRSA and vancomycin-resistant *S*. *aureus* (VRSA)) are responsible for a majority of infections associated with these devices, thereby leading to treatment failures. Once established, staphylococcal biofilms become resistant to antimicrobial treatment and host response, thereby serving as the etiological agent for recurrent infections. This study investigated the efficacy of octenidine hydrochloride (OH) for inhibiting biofilm synthesis and inactivating fully-formed staphylococcal biofilm on different matrices in the presence and absence of serum protein. Polystyrene plates and stainless steel coupons inoculated with *S*. *aureus*, MRSA or VRSA were treated with OH (zero, 0.5, one, 2 mM) at 37 °C for the prevention of biofilm formation. Additionally, the antibiofilm effect of OH (zero, 2.5, five, 10 mM) on fully-formed staphylococcal biofilms on polystyrene plates, stainless steel coupons and urinary catheters was investigated. OH was effective in rapidly inactivating planktonic and biofilm cells of *S*. *aureus*, MRSA and VRSA on polystyrene plates, stainless steel coupons and urinary catheters in the presence and absence of serum proteins. The use of two and 10 mM OH completely inactivated *S*. *aureus* planktonic cells and biofilm (>6.0 log reduction) on all matrices tested immediately upon exposure. Further, confocal imaging revealed the presence of dead cells and loss in biofilm architecture in the OH-treated samples when compared to intact live biofilm in the control. Results suggest that OH could be applied as an effective antimicrobial to control biofilms of *S*. *aureus*, MRSA and VRSA on appropriate hospital surfaces and indwelling devices.

## 1. Introduction

The Nosocomial Infections Surveillance System recognizes *Staphylococcus aureus* as the most frequently isolated nosocomial pathogen from patients [1]. Additionally, a high percentage of these isolates were found to be methicillin resistant (89% of identified *S*. *aureus* isolates). Methicillin-resistant *S*. *aureus* (MRSA) is the most commonly identified antibiotic resistant pathogen [2]. It is responsible for causing complicated skin and skin-structure infections and serious hospital-acquired infections [3]. Vancomycin has long been used as the antimicrobial agent for the treatment of MRSA infections in patients. However, this has led to the emergence of vancomycin-resistant *S*. *aureus* [4] (VRSA). It is estimated that staphylococci normally colonize 20%–25% of healthy adults permanently and 75%–80% transiently [5]. Millions of indwelling devices are implanted in patients every year, and staphylococci are responsible for a majority of infections and treatment failures linked to these devices [6]. Indwelling devices become coated with host-derived extracellular matrix proteins that provide a rich surface for bacterial attachment [7]. This ability to bind proteins facilitates pathogen attachment to plastic surfaces and other matrices [8]. Once established, staphylococcal biofilms are resistant to antimicrobial treatment and host response, besides serving as the etiological agent for recurrent infections [9]. Biofilm-associated staphylococci can lead to several diseases, including osteomyelitis, chronic wound infection endocarditis, polymicrobial biofilm infections and indwelling medical device infections [10].

The most commonly followed approach in the management of such infections is the removal and replacement of the contaminated devices [10]. An alternative to this is the use of antimicrobials or other technologies to prevent and control bacterial biofilms on indwelling devices. A variety of antimicrobials, including plant essential oils, phages, EDTA, nitric oxide, quorum sensing inhibitors and biofilm dispersants, such as oxidizing biocides, have been evaluated for controlling staphylococcal biofilms [10,11]. Although these approaches have shown promise in the control of staphylococcal biofilms, it is essential that these compounds maintain their efficacy in the presence of host proteins.

Octenidine hydrochloride (OH) is a positively-charged bispyridinamine exhibiting antimicrobial activity against plaque-producing organisms, such as *Streptococcus mutans* and *S*. *sanguis* [12]. Recent studies have also demonstrated its antimicrobial effect against *E*. *coli* O157:H7, *Salmonella* Enteritidis, *Acinetobacter baumannii*, *Candida albicans* and *Fusobacterium nucleatum*, *S*. *aureus* and *Pseudomonas aeruginosa* [13,14,15,16,17]. Toxicity studies in a variety of species have shown that OH is not absorbed through the mucous membrane and gastrointestinal tract, with no reported carcinogenicity, genotoxicity or mutagenicity [18].

The objective of this study was to investigate the efficacy of OH for inhibiting biofilm formation by *S*. *aureus*, MRSA and VRSA and inactivating pre-formed *S*. *aureus*, MRSA and VRSA biofilms at 37 °C in the presence and absence of serum proteins on polystyrene matrix, stainless steel coupons and urinary catheters.

## 2. Results and Discussion

OH was found to be equally effective against *S*. *aureus*, MRSA and VRSA biofilms. No significant differences were observed between the different isolates (*p* < 0.05). Therefore, the results obtained with one representative isolate of *S*. *aureus* (ATCC 35556), VRSA (VRS 8) and MRSA (NRS 123) are provided here. OH was found not only to be effective at killing planktonic cells and preventing biofilm formation, but also at inactivated fully established staphylococcal biofilms.

### 2.1. Prevention of Biofilm Formation

**Figure 1:**
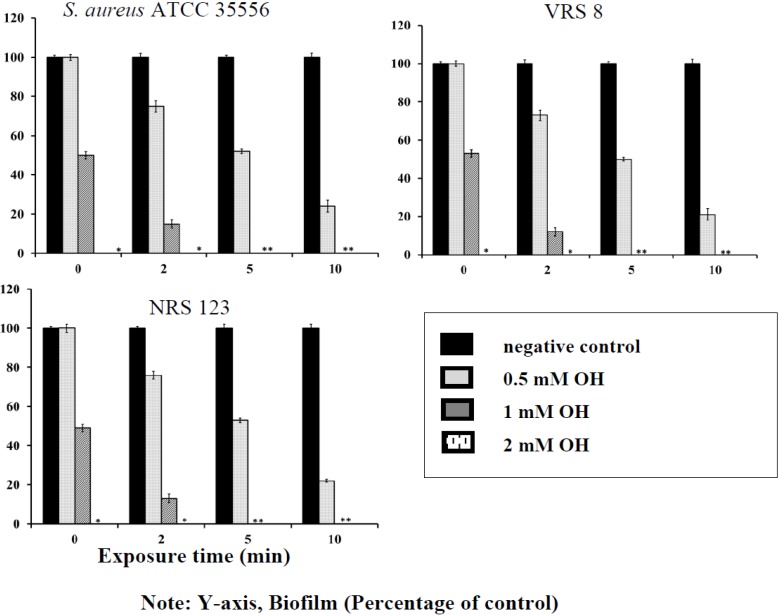
Inhibition of *S*. *aureus* (ATCC 35556), vancomycin-resistant *S*. *aureus* (VRSA) (VRS 8) and MRSA (NRS 123) biofilm formation on polystyrene by octenidine hydrochloride (OH). Duplicate samples were used for each treatment, and the experiment was replicated three times. Data are represented as the mean ± SEM.

**Figure 2:**
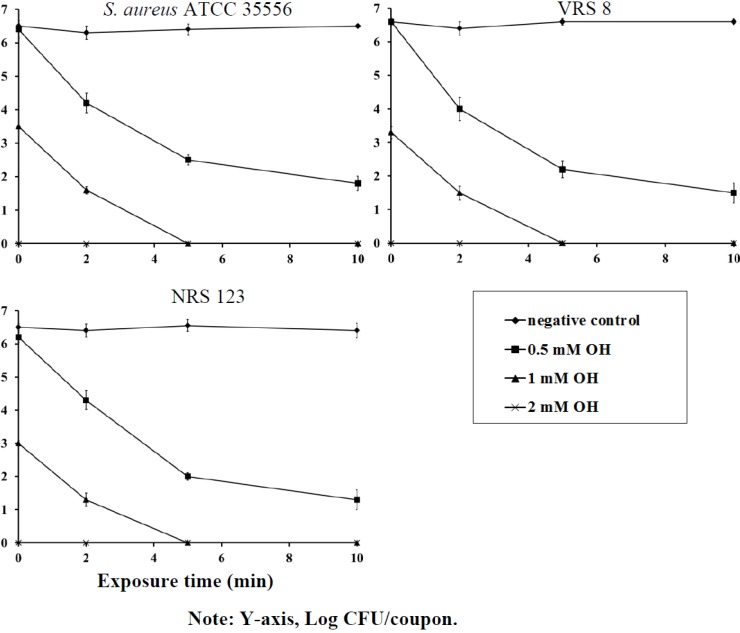
Inhibition of *S*. *aureus* (ATCC 35556), VRSA (VRS 8) and MRSA (NRS 123) biofilm on stainless steel by octenidine hydrochloride. Duplicate samples were used for each treatment, and the experiment was replicated three times. Data are represented as the mean ± SEM (Standard Error of Mean).

The efficacy of OH in preventing biofilm formation on polystyrene and stainless steel coupons is depicted in Figure 1 and Figure 2. OH was effective in rapidly inactivating planktonic staphylococci cells, thereby preventing the establishment of biofilms on polystyrene and stainless steel surfaces. With planktonic cells, 2 mM of OH completely inactivated staphylococcal populations immediately upon exposure, whereas 1 mM of OH reduced bacterial counts by greater than 3.0 log CFU/mL on contact (Figure 1 and Figure 2). As expected, staphylococcal populations in negative control samples remained the same throughout the sampling period. A set of samples were also assayed after 24 h to investigate biofilm formation. It was observed that the negative control samples had a fully formed biofilm, while the treated samples did not have any surviving population at 24 h (data not shown). When the efficacy of OH was tested for its ability to prevent biofilm formation in the presence of serum protein, OH retained its antimicrobial efficacy and resulted in a similar antibiofilm effect, as observed in the absence of protein (data not shown).

### 2.2. Inactivation of Established Biofilm

**Figure 3:**
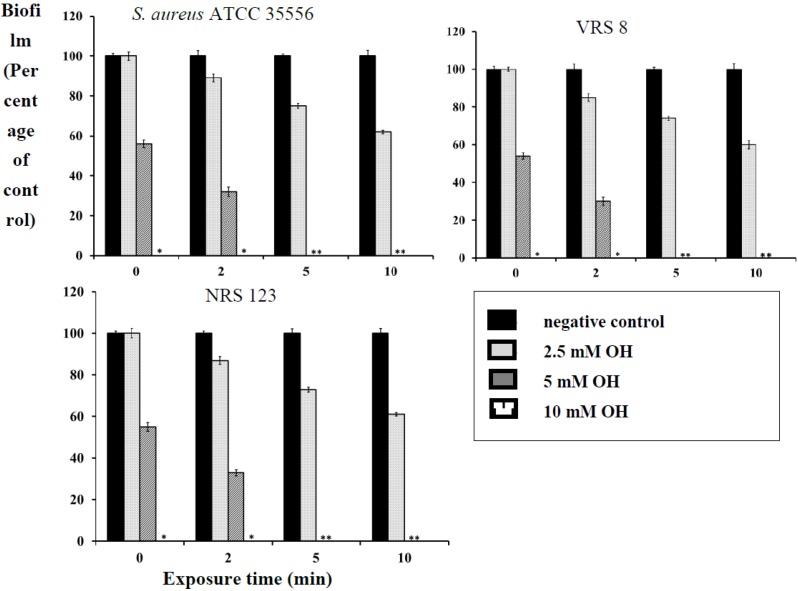
Inactivation of *S*. *aureus* (ATCC 35556), VRSA (VRS 8) and MRSA (NRS 123) biofilm on polystyrene by octenidine hydrochloride. Duplicate samples were used for each treatment, and the experiment was replicated three times. Data are represented as the mean ± SEM(Standard Error of Mean).

**Figure 4:**
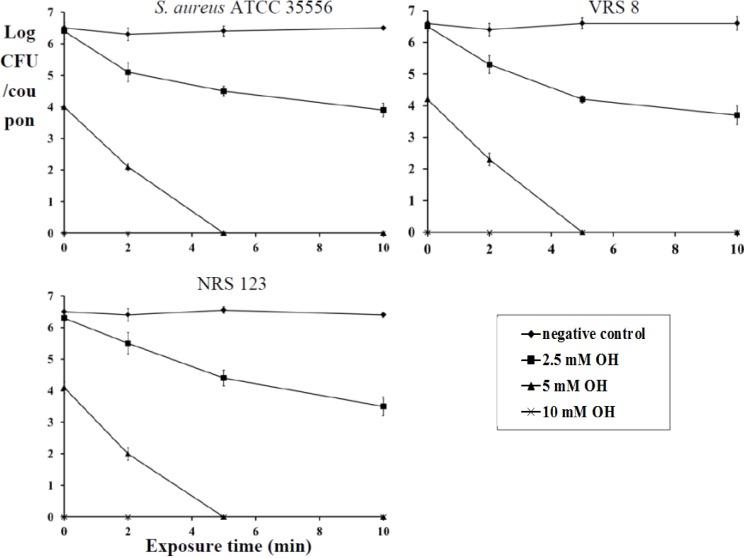
Inactivation of *S*. *aureus* (ATCC 35556), VRSA (VRS 8) and MRSA (NRS 123) biofilm on stainless steel by octenidine hydrochloride. Duplicate samples were used for each treatment, and the experiment was replicated three times. Data are represented as the mean ± SEM (Standard Error of Mean).

OH was also effective at killing fully formed biofilms of *S*. *aureus*, MRSA and VRSA on polystyrene and stainless steel (*p* < 0.05). Staphylococcus has been demonstrated to form biofilms on stainless steel implants, such as screws and fragment implants [19]. Therefore, the antibiofilm effect of OH was also investigated on a stainless steel matrix. At 10- and 5-mM levels, OH completely inactivated the biofilm immediately after addition (0 min) and 5 min of exposure, respectively (Figure 3 and Figure 4). As observed with planktonic cells, the biofilm inactivation by OH was not affected by the presence of serum albumin (data not shown). A similar reduction in biofilm populations was observed by Junka and others [16], who tested the antimicrobial efficacy of octenisept, a commercially available antiseptic that contains octenidine dihydrochloride. They observed a complete inactivation of the *S*. *aureus* biofilm on polystyrene discs within 1 min of contact time. Another study by Sennhenn-Kirchner [20] evaluated the antimicrobial efficacy of OH on biofilm formed by aerobic oral bacteria on rough titanium surfaces. Their study revealed that rinsing with OH for 8 min reduced the biofilm by 99.8%. However, our study demonstrates that exposure of the biofilm to 10 mM OH completely inactivated it immediately after addition. Besides biofilm inactivation, it is also interesting to note that the antibiofilm effect of OH was irrespective of the strains employed. It was equally effective on the antibiotic-resistant strains (MRSA and VRSA), especially in light of their association with nosocomial and community-acquired infections.

**Figure 5:**
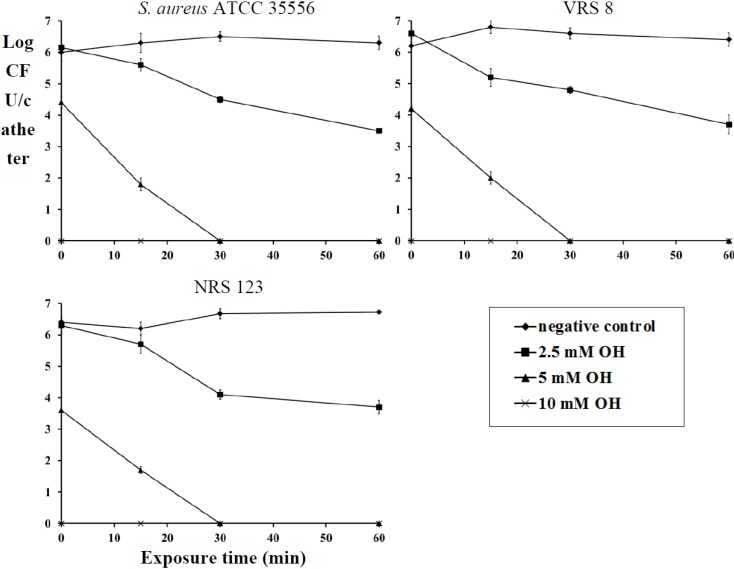
Inactivation of *S*. *aureus* (ATCC 35556), VRSA (VRS 8) and MRSA (NRS 123) biofilm on urinary catheters by octenidine hydrochloride. Duplicate samples were used for each treatment, and the experiment was replicated three times. Data are represented as the mean ± SEM (Standard Error of Mean).

Staphylococci have been shown to infect and form biofilms on orthopedic implants, stents, intravenous catheters, infusion pumps, mechanical heart valves, pacemakers and cosmetic surgical implants [19]. Treatment of these foreign-body-associated infections caused by MRSA and VRSA are difficult because of the limited availability of antibiotic options that are effective against bacterial biofilms [21]. The current treatment protocol against MRSA involves the use of vancomycin (plus rifampin when the bacteria are susceptible) [22]. However, the increase in the MICs of vancomycin and rifampicin needed for the treatment against MRSA and VRSA is of significant concern [23]. Chaudhury and others [24] investigated the ability of ethanol for the eradication of MRSA biofilms. They observed that the use of ethanol at a 40% concentration could inactivate the biofilms in 1 h. Although efficacious, there are several concerns regarding ethanol use. These include concerns about systemic exposure to ethanol, an increase in catheter dysfunction [25] and the effect of prolonged exposure to ethanol on catheter integrity, which have limited the widespread use of ethanol locks. Besides ethanol, recent study by Rosenblatt and others [26] demonstrated that the use of lock solution containing 7% citrate, 20% ethanol and 0.01% glyceryl trinitrate was able to inactivate MRSA biofilm in 2 h of exposure. Although these approaches have shown promise in the control of MRSA biofilms, it is essential that these compounds maintain their efficacy in the presence of host proteins. A study by Zumbotel and others [17] evaluated the ability of OH to prevent or delay *S*. *aureus* biofilm formation in OH-coated tracheostomy tubes. This study demonstrated that OH-coated tubes reduced the biofilm associated *S*. *aureus* population by 2 log compared to the negative control. However, reprocessing of the OH-coated tubes did not result in any significant reduction in biofilm formation. Therefore, in this present study, we investigated the antibiofilm effect of OH as a lock solution using urinary catheters as a model for indwelling devices. Inoculation of catheters with *S*. *aureus*, MRSA or VRSA resulted in a mature biofilm by the fifth day of incubation at 37 °C. A fully-formed staphylococcal biofilm was recovered from negative control catheters even after 24 h of incubation, whereas no biofilm was detected on catheters within 15 min of exposure to 10 mM of OH (Figure 5). After 60 min of exposure, 5 mM of OH also completely eliminated staphylococcal biofilms (Figure 5). However, staphylococcal biofilm counts on negative control catheters remained at 6.0 log CFU/mL throughout the experiment (Figure 5). A similar antibiofilm effect of OH was also observed in the presence of serum albumin. The ability of OH to retain its antibiofilm efficacy in the presence of serum albumin is of significance, since the presence of host proteins on the indwelling devices enhances the ability of pathogens to attach and form biofilms [1]. The antibiofilm effect of OH was compared with that of tetrasodium EDTA at a concentration of 40 mg/mL [11]. No significant decrease in staphylococcus populations in the biofilm was observed, even after an exposure time of 60 min to the EDTA (data not shown).

OH exerts its antimicrobial effect by binding to the negatively charged bacterial cell envelope, thereby disrupting the vital functions of the cell membrane and killing the cell [27]. It has a high affinity towards cardiolipin, a prominent lipid in bacterial cell membranes, making it selectively lethal to bacterial cells without adversely affecting eukaryotic cells [21]. In addition, Al-Doori and coworkers [28] reported that repeated exposure of *S*. *aureus* to OH for up to three months did not induce resistance to the compound.

### 2.3. Confocal Microscopy

**Figure 6:**
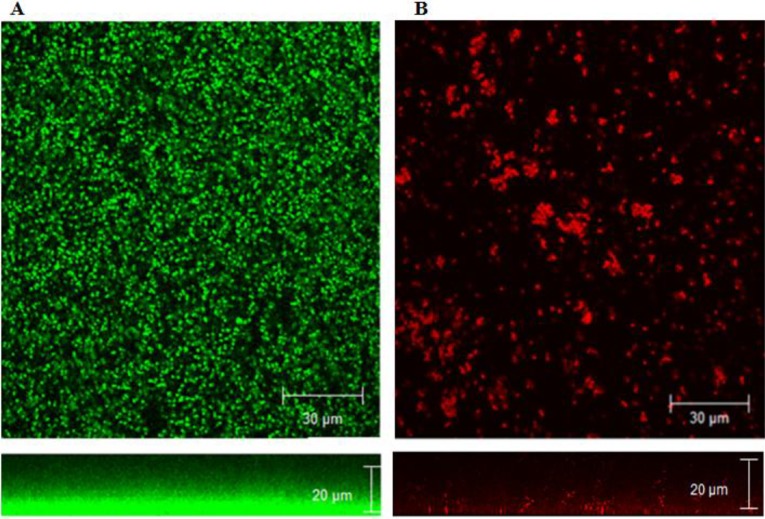
Confocal microscopy of MRSA (NRS 385) biofilm without treatment (**A**) and after treatment with octenidine hydrochloride (**B**).

To investigate the effect of OH on biofilm structure, staphylococcus biofilms formed on glass coverslips were analyzed by confocal microscopy. Positive staining using SYTO^®^ (Green fluorescent nucleic acid stain) and propidium iodide (PI) was used for the imaging. The confocal images of the negative control biofilm (Figure 6A) with no added OH revealed the formation of dense biofilm (average thickness 15 µm) viewed as green cells (live) stained by the SYTO dye, while the image of OH-treated samples (Figure 6B) revealed patchy breaks in biofilm due to the loss of cells and the disruption of organization, viewed as red cells (dead) stained by PI. The average thickness of OH-treated biofilms was 1 µm. These data collectively indicate that OH was effective at preventing biofilm formation by *S*. *aureus*, MRSA and VRSA, as well as rapidly inactivating pre-formed biofilms on polystyrene, stainless steel and urinary catheters.

## 3. Experimental Section

### 3.1. Culture Preparation

All bacteriological media were purchased from Difco (Becton Dickinson, Sparks, MD, USA). The antibiofilm effect of OH was investigated on *S*. *aureus* (ATCC 35556, and ATCC 12600), methicillin-resistant *S*. *aureus* (MRSA; NRS 123, NRS 385, NRS 194) and vancomycin-resistant *S*. *aureus* (VRSA; VRS 8, VRS 9, VRS 10). MRSA and VRSA strains were obtained from the Network on Antibiotic Resistant *Staphylococcus aureus* (NARSA, Chantilly, VI, USA). Stock cultures were stored at −80 °C in brain heart infusion broth (BHI) with 50% glycerol. Prior to each experiment, a loopful of culture was grown in 10 mL of BHI with incubation at 37 °C for 24 h. The culture was sedimented by centrifugation (3600× g, 12 min, at 4 °C), washed and resuspended in phosphate buffered saline (1× PBS pH 7.2 consisting of 137 mM NaCl, 2.7 mM KCl, 10 mM Na_2_HPO_4_ and 2 mM KH_2_PO_4_) and used as the inoculum. The bacterial count of the inoculum was determined by plating on BHI agar plates and incubation at 37 °C for 24 h.

### 3.2. Octenidine Hydrochloride

OH (>99%) was obtained from Dishman USA, Middlesex, NJ, USA.

### 3.3. Prevention of Biofilm Formation by S. aureus, MRSA and VRSA on Polystyrene by OH

The efficacy of OH in inhibiting *S*. *aureus*, MRSA and VRSA biofilm production was investigated according to the method of Amalaradjou *et al.* [29]. Briefly, *S*. *aureus*, MRSA and VRSA strains were separately grown overnight in BHI at 37 °C. Following incubation, the cultures were sedimented by centrifugation (3,600× g for 15 min), washed twice with PBS and resuspended in 10 mL of BHI. Two hundred microliters of the washed culture were used as the inoculum (~6.0 log CFU). Sterile 96-well polystyrene tissue culture plates (Falcon, Franklin lakes, NJ) were inoculated with 200 µL of bacterial suspension, followed by the addition of 0 (negative control), 0.5 (0.25 μL), 1 (0.5 μL) or 2 (1 μL) mM OH (dissolved in 95% ethanol). The plates were incubated at 37 °C. Following 0, 2, 5 and 10 min of OH exposure, the surviving bacterial populations were enumerated by serial dilution (1:10 in PBS) and plating on duplicate BHI plates. When *S*. *aureus* was not detected by direct plating, samples were tested for surviving cells by enrichment at 37 °C for 24 h in 100 mL of BHI, followed by streak plating on mannitol salt agar (MSA). Representative colonies on BHI were confirmed as staphylococci based on colony morphology on MSA. Triplicate samples were included for each treatment, and the experiment was replicated three times.

### 3.4. Inactivation of S. aureus, MRSA and VRSA Biofilms Formed on Polystyrene by OH

The antibiofilm effect of OH was determined by microtiter plate assay [29]. Sterile 96-well polystyrene tissue culture plates (Costar, Corning Incorporated, Corning, NY, USA) were inoculated with 200 µL of the each bacterial cell suspension (~6.0 logCFU) and incubated at 37 °C for 24 h without agitation for biofilm production. Following biofilm formation, the effect of OH was tested at 0 (negative control), 2.5 (1.5 µL), 5 (2.5 µL) and 10 (5 µL) mM concentrations with an exposure time of 0, 2, 5 and 10 min. After exposure to OH for the specified time, the wells were washed three times with 200 µL of sterile PBS, dried at room temperature and finally stained with 1% crystal violet for 15 min. After rinsing three times with sterile distilled water and subsequent destaining with 95% ethanol, the absorbance of the adherent biofilm was measured at 570 nm in a microplate reader (Model 550, Bio-Rad, Hercules, CA, USA). Uninoculated wells containing BHI were used as blanks. Blank-corrected absorbance values were used for reporting biofilm production. Five replicate wells were included for each treatment, and the assay was repeated three times.

### 3.5. Enumeration of Bacterial Counts in Biofilm

In addition to the microtiter plate assay, the antibiofilm effect of OH was also assayed by enumerating surviving bacterial populations in the biofilm using the viable plate count method [29]. Following exposure to OH, the wells were washed three times with PBS, and the adherent biofilm was scraped and plated directly or after serial dilution in PBS on BHI plates. The plates were incubated at 37 °C for 24 h before enumerating the bacterial colonies.

### 3.6. Biofilm Assay on Stainless Steel Matrix

Stainless steel (type 304 with a 4b finish) was used for making coupons (diameter: 1 cm) [30]. Stainless steel coupons were washed and cleaned prior to use, as described by Amalaradjou *et al.* [27].

#### 3.6.1. Biofilm Assay

*S*. *aureus*, MRSA and VRSA cells were grown and diluted 1:40, as described before. Two hundred microliters of the inoculum were then dispensed onto the stainless steel coupons submerged in a 24-well plate (Falcon, Becton Dickson Labware, Franklin Lakes, NJ, USA). Biofilm was formed at 37 °C, as before, and treated with 0 (negative control), 2.5, 5 and 10 mM of OH for an exposure time of 0, 2, 5 or 10 min. A procedure described by Ayebah and coworkers [30] was used to remove, disperse and enumerate the cells in biofilm. Duplicate coupons were included for each treatment, and the experiment was replicated three times.

### 3.7. Biofilm Assay on Catheters

The efficacy of OH for inactivating fully-formed *S*. *aureus*, MRSA and VRSA biofilms on catheters was determined according to a previously described protocol [29]. A latex 12 F Foley urinary tract catheter (AtHomeMedical) was cut into 3-cm pieces. Each catheter piece was sealed at one end, filled with 1 mL of bacterial culture (~6.0 log CFU) and sealed at the other end. The catheter pieces were then incubated at 37 °C for 5 days to facilitate biofilm formation onto the catheter lumen surface. After 5 days, each catheter piece was washed with sterile saline to remove unattached cells, sealed at one end, filled with 1 mL of sterile normal saline (negative control) or saline containing 2.5, 5 and 10 mM of OH, sealed at the other end and incubated at 37 °C. The biofilm-associated bacterial population was determined following OH exposure (0, 15, 30 and 60 min) by enumerating bacteria after dislodging the biofilm from the catheter surface. This was achieved by vortexing the catheter pieces in separate tubes containing 10 mL of PBS for 1 minute, followed by sonication at 40 KHz for 5 min in a bath sonicator (Branson, North Olmstead, OH, USA). After sonication, viable bacterial counts in PBS from each tube were enumerated after serial dilution (1:10 in PBS) and plating on duplicate BHI plates. Three catheter pieces were included, and the experiment was repeated three times.

### 3.8. Antibiofilm Effect of OH in the Presence of Serum Protein

The efficacy of OH for inhibiting and inactivating the biofilm of *S*. *aureus*, MRSA and VRSA in the presence of serum protein was determined according to the method of Edmiston and others [31]. Rehydrated bovine serum albumin (20%) was used to simulate the presence of proteins on indwelling devices. To determine the efficacy of OH in preventing *S*. *aureus*, MRSA and VRSA biofilm from planktonic cells in the presence of serum proteins, bovine serum albumin was added to each well/stainless steel coupon prior to microbial challenge followed by OH addition (0 (negative control), 0.5, 1 or 2 mM) for 0, 1, 2 and 5 min at 37 °C. Following exposure to OH, the surviving population of bacteria was enumerated by the viable plate count, as described previously. Three samples were included for each treatment, and the assay was replicated three times. For determining the efficacy of OH for killing established bacterial biofilms in the presence of proteins, biofilms were grown in the presence of bovine serum albumin on the different matrices tested and exposed to OH (0 (negative control), 2.5, 5 and 10 mM)| for 0, 2, 5 and 10 min on polystyrene and stainless steel and 0, 15, 30 and 60 min on catheters. The biofilms were assayed, as described before. This study was done at 37 °C. Five replicate wells were included for each treatment, and the assay was repeated three times.

### 3.9. Confocal Microscopy

To obtain depth-selective information on the three-dimensional structure of the biofilm, *in situ* confocal laser scanning microscopy was performed. For microscopic assessment, biofilms were grown at 37 °C in BHI on a Lab-Tech 8-chambered #1 borosilicate cover glass system (Lab-Tek, Nalge Nunc International, Rochester, NY, USA). The microscopy was performed according to the method reported by Amalaradjou *et al.* [29]. The biofilms formed on cover slips were treated with OH (10 mM), and the live and dead cells were imaged after staining with 2.5 µM SYTO (Molecular probes, OR) and 5 µMpropidium iodide (PI, Molecular probes, OR). Biofilms not exposed to OH (negative control) were also imaged to view the normal architecture of *S*. *aureus*, MRSA and VRSA biofilm. Samples were examined under a Leica true confocal scanner SP2 microscope using the water immersion lens. A krypton-argon mixed gas laser with a PMT2 (Photomultiplier tube 2) filter served as the excitation source.

### 3.10. Statistical Analysis

Duplicate samples were used for each treatment, and each experiment was replicated three times. For each treatment and the control, the data from independent replicate trials were pooled and analyzed using the proc mixed sub-routine of the statistical analysis software. The model included the treatment concentrations and time as the major effects. A least significant difference test was used to determine significant differences (*p* < 0.05) due to treatment concentrations and time on bacterial counts.

## 4. Conclusions

In conclusion, our study demonstrates that OH was effective in preventing biofilm formation by *S*. *aureus*, MRSA and VRSA and rapidly inactivating pre-formed biofilms on polystyrene, stainless steel and urinary catheters. In addition, OH was equally effective against biofilms in the presence and absence of serum proteins. These results suggest that OH can be potentially used as a sanitizer for hospital surfaces. Since *S*. *aureus*, MRSA and VRSA have the ability to persist in the hospital environment and form biofilms on a wide variety of fomite surfaces, OH can be used as a potential antimicrobial lock solution in both treatment and prophylactic modalities. However, further experiments are needed to further evaluate the efficacy of OH in comparison with other anti-MRSA therapies *in vitro* and *in vivo*. Along with improvements in catheter design and coating, the universal adoption of strict aseptic techniques and the appropriate use of novel catheter lock solutions, such as OH, that minimize catheter-related infections may help to decrease the morbidity and mortality associated with foreign-body-associated infections.
